# The impact of social norms interventions on clinical behaviour change among health workers: protocol for a systematic review and meta-analysis

**DOI:** 10.1186/s13643-019-1077-6

**Published:** 2019-07-18

**Authors:** Sarah Cotterill, Rachael Powell, Sarah Rhodes, Benjamin Brown, Jane Roberts, Mei Yee Tang, Jack Wilkinson

**Affiliations:** 1 Centre for Biostatistics, Division of Population Health, Health Services Research and Primary Care, School of Health Sciences, Faculty of Biology Medicine and Health, University of Manchester, Oxford Road, Manchester, M13 9PL UK; 2 Manchester Centre for Health Psychology, Division of Psychology and Mental Health, School of Health Sciences, Faculty of Biology Medicine and Health, University of Manchester, Manchester, UK; 3 Health e-Research Centre, Farr Institute for Health Informatics Research, Faculty of Biology Medicine and Health, University of Manchester, Manchester, UK; 4 Centre for Primary Care, School of Health Sciences, Faculty of Biology Medicine and Health, University of Manchester, Manchester, UK; 5 Outreach and Evidence Search Service, Library & E-learning Service, The Pennine Acute Hospitals NHS Trust, Royal Oldham Hospital, Oldham, UK

**Keywords:** Systematic review, Meta-analysis, Social norm, Social comparison, Information about others’ approval, Credible source, Social reward, Social incentive, Feedback, Behaviour change

## Abstract

**Background:**

Health workers routinely carry out clinical behaviours, such as prescribing, test-ordering or hand-washing, which impact on patient diagnoses, care, treatment and recovery. Social norms are the implicit or explicit rules that a group uses to determine values, beliefs, attitudes and behaviours. A social norms intervention seeks to change the clinical behaviour of a target health worker by exposing them to the values, beliefs, attitudes or behaviours of a reference group or person. This study aims to find out whether or not social norms interventions are effective ways of encouraging health workers to carry out desired behaviours and to identify which types of social norms intervention, if any, are most effective.

**Methods:**

A systematic review will be conducted. The inclusion criteria are a population of health professionals, a social norms intervention that seeks to change a clinical behaviour, and randomised controlled trials. Searches will be undertaken in MEDLINE, EMBASE, CINAHL, British Nursing Index, ISI Web of Science, PsycINFO and Cochrane trials. Titles and abstracts will be reviewed against the inclusion criteria to exclude any that are clearly ineligible. Two reviewers will independently screen all the remaining full texts to identify relevant papers. For studies which meet our inclusion criteria, two reviewers will extract data independently, code for behaviour change techniques and assess quality using the Cochrane risk of bias tool. The primary outcome measure will be compliance with desired behaviour. To assess the effect of social norms on the behaviour of health workers, we will perform fixed effects meta-analysis and present forest plots, stratified by behaviour change technique. We will explore sources of variation using meta-regression and may use multi-component-based network meta-analysis to explore which forms of social norms are more likely to be effective, if our data meet the necessary requirements.

**Discussion:**

The study will provide evidence regarding the effectiveness of different methods of applying social norms to change the clinical behaviour of health professionals. We will disseminate the research to academics, health workers and members of the public and use the findings from the review to plan future research on the use of social norms with health workers.

**Systematic review registration:**

PROSPERO CRD42016045718. Future protocol changes will be clearly stated in PROSPERO.

**Electronic supplementary material:**

The online version of this article (10.1186/s13643-019-1077-6) contains supplementary material, which is available to authorized users.

## Background

Health workers routinely carry out behaviours which impact on patient diagnoses, care, treatment and recovery. Many of these behaviours have clear guidelines for best practice. Examples include appropriate ordering of diagnostic tests [1, 2], appropriate prescription of antibiotics [3, 4], regular recall of patients with long-term conditions [5], hand-washing [6] and choice of wound dressings [7]. Health workers face many challenges in following evidence-based professional practice. There is evidence that social influences are important in clinical practice [8, 9].

One proposed solution has been to implement behaviour change interventions based on social or peer norms. Social norms are the implicit or explicit rules that a group uses to determine values, beliefs, attitudes and behaviours. A social norms intervention seeks to change the clinical behaviour of a target health worker by exposing them to the values, beliefs, attitudes or behaviours of a reference group or person. These social norms interventions can form part of an audit and feedback initiative [10–12] or may be developed as another behaviour change intervention [13]. These are interventions with reach that can be implemented routinely across multiple health workers and settings at low cost, so the absolute gain can be very large. We use the term target to refer to a health worker who is targeted by social norms interventions, with a view to changing their clinical behaviour. We use the term reference group or reference person to mean a person or group of people used as a reference category in a social norms intervention. For the purposes of our review, we anticipate that reference categories will include people with the same profession or occupation as the target; people employed by the same organisation as the target; people who deliver, administer, manage, commission or make policy on health services; or professional bodies such as royal colleges and trade unions. It is possible that some studies will use social norms approaches where the reference group is not taken from the above list (such as credible source from a celebrity or exposing the target’s behaviour to patients). We will include in the review papers with any type of reference group.

The ability of social norms to affect behaviour has been considered within several behaviour change theories and theoretical frameworks. For example, ‘subjective norm’ is a construct within the Theory of Planned Behaviour [14], which describes an individual’s perceptions of whether valued others think one should perform a behaviour, combined with one’s motivation to comply with others’ beliefs. The Theory of Normative Social Behaviour [15] proposes that behaviour can be changed through normative mechanisms and has made distinctions between descriptive norms (beliefs concerning the prevalence of a behaviour) and injunctive norms (beliefs concerning what one feels they ought to do based on others’ expectations—social approval). Further, the ‘social influences’ domain of the Theoretical Domains Framework [16] also includes several normative constructs: social norms, social comparisons and group norms. We will include studies based on either descriptive norms or injunctive norms messages. A descriptive norms message provides the target with information about the behaviour of others in the reference group. Examples of descriptive norms interventions include giving the target information about the behaviour of a reference person or group or comparing the target’s behaviour with the behaviours of a reference person or group. An injunctive norms message provides the target with information about the values, beliefs or attitudes of the reference group, conveying social approval or disapproval. Examples of injunctive norms interventions include providing the target with information about whether the behaviour has the approval/disapproval of the reference group or person, exposure (actual or promised) of the target’s behaviour to a reference group and praise, commendation, applause or thanks (actual or promised) from a reference group or person.

The behaviour change technique taxonomy v1 is a list of 93 distinct behaviour change techniques (BCTs) which are used in behaviour change interventions [17]. The BCT Taxonomy includes five BCTs which we believe involve social norms: social comparison, information about others’ approval, credible source, social reward and social incentive [17]. We have chosen to define social norms in terms of the BCT taxonomy v1 because, based on international consensus, it aims to define and label all active ingredients of interventions, including social norms. It incorporates previous behaviour change taxonomies and has involved significant effort from leaders in the field and considerable investment from the MRC and NIHR in developing the taxonomy. We believe this to be the most reliable tool currently available that can define BCTs. We have selected the five BCTs that we consider have a social norms element to them, and we have discussed this selection carefully, both within the research team and with our steering group of international experts. We are open to the possibility that studies may be eligible for the review that test social norms interventions but do not incorporate one of these five identified BCTs.

Health workers frequently receive audit and feedback (A&F), which involves ‘providing a recipient with a summary of their performance over a specified period of time’ ([10] p. 1). Social norms interventions are sometimes included as one component of A&F, such as when the health worker is shown information about their own performance and also a comparison with their peers [11, 12]. A&F has already been shown to be effective in changing health worker behaviour, but with large variation in outcomes depending on the context and the intervention design [18]. There is a need to understand the ingredients for successful A&F [10, 19], and the effects or mechanisms of the social norms constituents of A&F have been identified in a recent systematic review as topics for further research [10]. Our review will contribute to this important research agenda by systematically examining the evidence for using social norms BCTs with health workers.

### Aims

The overall aim is to conduct a systematic review to assess, among health workers, the impact of social norms BCTs, compared to alternative interventions, no intervention or comparison of one or more social norms BCTs on compliance with evidence-based professional practice. The review will address two research questions:

1. What is the effect of social norms interventions on the clinical behaviour of health workers and resulting patient outcomes?
2. Which contexts, modes of delivery and behaviour change techniques are associated with the effectiveness of social norms interventions on health worker clinical behaviour change?

## Methods

This protocol follows the PRISMA-P reporting guidelines for systematic reviews [20] (PRISMA-P checklist included as Additional file 1).

### Eligibility criteria

The inclusion criteria for the review are a population of health professionals, a social norms intervention that seeks to change a clinical behaviour, and the study type is a randomised controlled trial.

#### Population

The population of interest is health workers and managers. Student health workers will be included, but only if the study is in a healthcare setting. Any healthcare setting will be eligible, including care homes, nursing homes and patients’ own homes. Interventions in educational establishments or simulated environments will not be eligible.

#### Interventions

The systematic review will focus on social norms interventions, defined as interventions seeking to change the clinical behaviour of a target health worker by exposing them to the values, beliefs, attitudes or behaviours of a reference group or person. We have selected five BCTs (from the BCT taxonomy v1) that we consider have a social norms element to them (6.2. Social comparison; 6.3. Information about others’ approval; 9.1 Credible source; 10.4 Social reward; 10.5 Social incentive), but we are open to the possibility that studies may be eligible for the review that test social norms interventions without using one of the five BCTs. Three BCTs are used unchanged in this review (social comparison, information about others’ approval and credible source). Two BCTs have been adapted slightly for clarity: the definitions of social reward as ‘verbal or non-verbal reward’ and social incentive as ‘verbal or non-verbal incentive’ are insufficient to distinguish a ‘social’ reward incentive from other types of reward or incentive. Further, in the present study, we are interested in only those social rewards or incentives that rely on social norms. We define social reward and incentive as involving praise, commendation, applause or thanks, all of which are injunctive norms messages, providing the target with information about the values, beliefs or attitudes of the reference group, conveying social approval or disapproval (Table 1).

**Table 1:** Social norms BCTs for inclusion in the review

Name and Definition from BCT Taxonomy [17]	SOCIAL review name and definition
*6.2. Social Comparison* Draw attention to others’ performance to allow comparison with the person’s own performance. Note: being in a group setting does not necessarily mean that social comparison is actually taking place. Example: Show the doctor the proportion of patients who were prescribed antibiotics for a common cold by other doctors and compare with their own data.	*6.2. Social Comparison—unchanged*
*6.3. Information about others’ approval* Provide information about what other people think about the behaviour. The information clarifies whether others will like, approve or disapprove of what the person is doing or will do. Example: Tell the staff at the hospital ward that staff at all other wards approve of washing their hands according to the guidelines.	*6.3. Information about others’ approval—unchanged*
*9.1. Credible source* Present verbal or visual communication from a credible source in favour of or against the behaviour. Note: code this BCT if source generally agreed on as credible, e.g. health professionals, celebrities or words used to indicate expertise or leader in field and if the communication has the aim of persuading. Example: Present a speech given by a high status professional to emphasise the importance of not exposing patients to unnecessary radiation by ordering X-rays for back pain.	*9.1. Credible source—unchanged*
*10.4. Social reward* Arrange verbal or non-verbal reward if and only if there has been effort and/or progress in performing the behaviour (includes ‘Positive reinforcement’). Example: Congratulate the person for each day they eat a reduced fat diet.	*10.4. Social reward—changed* Arrange praise, commendation, applause or thanks if and only if there has been effort and/or progress in performing the behaviour (includes ‘Positive reinforcement’). Example: Arrange for a family doctor to be sent a thank you note for each week that they reduce their level of antibiotic prescribing. Reason for change: the definition of social reward as ‘verbal or non-verbal reward’ is insufficient to distinguish a ‘social’ reward from other types of reward. Further, in the present study, we are interested in only those social rewards that rely on social norms. Praise, commendation, applause or thanks are all injunctive norms messages, providing the target with information about the values, beliefs or attitudes of the reference group, conveying social approval or disapproval.
*10.5 Social incentive* Inform that a verbal or non-verbal reward will be delivered if and only if there has been effort and/or progress in performing the behaviour (includes ‘Positive reinforcement’). Example: Inform that they will be congratulated for each day that they eat a reduced fat diet.	*10.5 Social incentive—changed* Inform that praise, commendation, applause or thanks will be delivered if and only if there has been effort and/or progress in performing the behaviour (includes ‘Positive reinforcement’). Example: Promise a family doctor in advance that they will be sent a thank you note for each week that they reduce their level of antibiotic prescribing. Reason for change: the definition of social reward as ‘verbal or non-verbal reward’ is insufficient to distinguish a ‘social’ reward from other types of reward. Further, in the present study, we are interested in only those social rewards that rely on social norms. Praise, commendation, applause or thanks are all injunctive norms messages, providing the target with information about the values, beliefs or attitudes of the reference group, conveying social approval or disapproval.

Included studies must state a behaviour that is being targeted for change. By definition, BCTs relate to behaviour(s): ‘a single action or sequence of actions’. Either the ‘performance of wanted behaviour(s) and/or inhibition (non-performance) of unwanted behaviour(s)’ might be addressed by a BCT [17] (detail/quotes are from electronic supplementary materials, p1.). We will report the number of studies which would otherwise meet our inclusion criteria but do not mention a target behaviour.

The format of the behaviour change intervention may be letter, electronic or verbal. It may be delivered once only, repeated over time or delivered in a timely fashion on occasions when the behaviour is expected to be performed. For example, an intervention to reduce prescribing of antibiotics by family doctors might be delivered once only, by regular weekly email, or by a computerised reminder when a relevant disease code is entered into the practice computer system.

#### Comparators

We anticipate finding a range of comparators, including alternative intervention, no intervention or comparison of one or more social norms BCTs (Table 2).

**Table 2:** Types of comparison

	Interventions		Controls
1	Social norm intervention	vs	Any control
2	Social norm intervention + X	vs	X
3	Social norm intervention + X	vs	Any control
4	Social norm intervention + X	vs	Social norm intervention
5	Social norm intervention A	vs	Social norm intervention B

Where X is any other intervention and A and B are two different types of social norm behaviour change technique.

#### Study designs

The systematic review will only include randomised controlled trials (RCTs) of any design (cluster, factorial, parallel, cross-over and stepped wedge). The justification for restricting the review to RCTs is that the review is concerned with the effectiveness of social norms, and randomised controlled trials are the best method for assessing the effectiveness of an intervention. We will include both published and unpublished research. Studies must be reported in English because the research team has no resource for translation from other languages.

### Information Sources and search strategy

The search strategy was developed collaboratively between the researchers and the information specialist in our review team. The search targeted databases relevant to health, social and behavioural science, without restriction on dates: MEDLINE, Ovid; EMBASE, Ovid; CINAHL, Ebsco; British Nursing Index; ISI Web of Science; PsycINFO and Cochrane trials. The search was structured to find the populations (health workers), interventions (social norms) and study types (RCTs) of interest. The population search terms were based on health worker search terms from previous reviews, supplemented by a list of health worker roles from a local NHS trust and review by the study team. Intervention search terms were based on the descriptions of social norms BCTs in the BCT taxonomy v1, audit and feedback search terms, and theories relevant to social norms. RCT search filters are those described in Chapter 6.4,11 of the Cochrane Handbook for Systematic Reviews of Interventions [21]. The search terms were developed by the study team and used to develop searches in MEDLINE. They were then reviewed by the project team and translated into the other databases using the appropriate controlled vocabulary as applicable. The final search strategy was reviewed by the Management Group and Steering Group. Searches were completed between 4 and 19 July 2018. The MEDLINE search is available within our PROSPERO registration [22] and as Additional file 2.

### Data collection

#### Data management

Covidence will be utilised as a management tool for the review (https://www.covidence.org/).

#### Study selection process

One reviewer will independently screen the titles and abstracts and exclude studies which obviously do not meet inclusion criteria. A second reviewer will independently screen a sample of 20% of records. If there is any difference of opinion at the title/abstract stage, the reviewers will err on the side of inclusion. The number of cases of disagreement will be reported. If the level of disagreement is over 10%, all the records will be double screened. Two reviewers will independently screen the full texts and apply the eligibility criteria. If there is any difference of opinion, the two screeners will discuss, and if they cannot agree, the text will be reviewed by a third member of the research team or resolved at a project team meeting if required.

#### Data collection process

Data from included studies will be extracted independently by two researchers. A data collection template, based on the Cochrane EPOC data collection form [23] has been developed [22], which will ensure that (where available) information on population and setting, methods, participants, interventions, controls, outcomes, results and applicability is collected. Discrepancies between reviewers will be resolved through discussion between the two researchers, by involving a third member of the research team, or discussion within the full project team meeting where necessary. Interventions will be described using relevant items from the TIDieR checklist [24] and specific behaviour change techniques will be classified using the behaviour change technique taxonomy v1 [17].

#### Unit of analysis issues

If any of the studies in the review are cluster randomised trials, summary measures (e.g. means, odds ratios) and adjusted standard errors will be extracted from appropriately analysed trials. Where necessary, adjustments for clustering will be made, using the ICC (intra-class correlation coefficient). If more than one comparison from a study with more than two arms is eligible for the same comparison, the number of health workers in the shared arm will be adjusted to avoid double counting. The adjustment will be done by dividing the number of health workers in the shared arm approximately evenly among the comparisons.

#### Missing data

The research team will search for companion papers (by author searching and citation searching) and/or contact trial authors once to obtain missing information under the following circumstances:

1. Where there is insufficient information in the full trial report to establish whether or not the trial meets our inclusion criteria
2. Where it is clear that our primary outcome measure was measured but insufficient information was reported to establish the number of participants and/or summary measures
3. Where the intervention descriptions are insufficiently clear to determine whether or not the trial meets our inclusion criteria

The research team will impute estimates of standard deviations where necessary (after contacting authors) using standard deviations from other similar studies (same target behaviour, same setting) that use the same type of outcome. Where necessary, for cluster randomised trials, a value of the ICC from similar studies (same target behaviour, same outcome measure, same setting) will be imputed.

#### Piloting

All processes for screening, extraction, BCT coding and assessment of bias will be piloted within the team prior to implementation.

#### Coding of Behaviour Change Techniques

Specific behaviour change techniques will be coded independently by two researchers, using the behaviour change technique taxonomy v1 [17]. The reliability of identifying and coding behaviour change techniques from intervention descriptions has been assessed [25]. This research assessed the reliability of judgements between different coders and across time using the prevalence and bias-adjusted kappa (PABAK) statistic. Overall, there were high rates of reliability between coders. Of the five BCTs relevant to this review, three of the techniques (social comparison, information about others’ approval, social incentive) were assessed in a high number of studies (6 to 20 studies) and were found to have high inter-rater reliability (over 0.7). Social reward was assessed in only one study but the reliability was high (1.0). Credible source was found in a high number of studies but found to have lower reliability (0.4) (Table 3).

**Table 3:** Inter-rater reliability of coding of social norms BCTs

	Behaviour change technique	Number of studies in which the BCT was present	Inter-rater reliability (PABAK)
6.2	Social comparison	13	0.76
6.3	Information about others’ approval	6	0.94
9.1	Credible source	32	0.4
10.4	Social incentive	7	0.9
10.5	Social reward	1	1.0

Source: [24]

All coders will be trained in coding of behaviour change techniques, using the on-line training provided by the authors of the BCT taxonomy (http://www.bct-taxonomy.com/), which is required to meet acceptable standards of competence. The online training has been shown to improve agreement with expert consensus, confidence for BCTs assessed and coding competence [26]. Additionally, they have attended a workshop facilitated by co-applicant RP and steering group member MJ. The level of agreement on the coding of BCTs between two coders will be reported, using a PABAK statistic.

#### Outcomes and prioritisation

The primary outcome for this review is compliance of the health worker with the desired clinical behaviour (e.g. rate of antibiotic use) at 6 months post randomisation. Six months post randomisation was chosen to identify a common time point from randomisation, and there is the potential for interventions to run over several months. Researchers will extract details of the outcome closest to 6 months post randomisation (e.g. mean and standard deviation or proportion). Other time points will be noted for inclusion in the description of studies and the team may consider conducting analysis using earlier/later time points, where reported.

It is likely that different studies will use different outcome measures as they will be measuring different behaviours and using different methods to assess those behaviours. The research team will convert any observed measure of health worker behaviour into a standardised mean difference between groups in terms of compliance with the desired behaviour. Examples include the mean number of times the behaviour was performed per worker or the mean rate of behaviour (e.g. rate of antibiotic items dispensed per 1000 population). It is possible that compliance will be reported as a binary outcome, for example compliance vs non-compliance on a single occasion, e.g. attendance a training session. The methods of Chin 2000 will be adopted to convert binary outcomes to standardised mean differences with associated standard errors using the formula [27]:

$$ \mathrm{SMD}=\frac{\sqrt{3}}{\pi}\ln \mathrm{OR} $$

If several measures of compliance are reported in a trial, the following criteria will be used to select the outcome for the primary analysis, in decreasing order of importance: (a) observed measure rather than self-report, (b) continuous measure, (c) final score rather than change from baseline or percentage change, (d) described as the primary outcome, (e) used to calculate the sample size, and (f) reported first.

A secondary outcome for the review is any patient outcomes which are likely to result from targeting the health worker behaviour.

#### Risk of bias in individual studies

Two reviewers will independently assess the risk of bias of each study; discrepancies will be resolved by discussion between the two reviewers, by involving a third reviewer, or by discussion within the project team, if needed. The risk of bias for each main outcome in all studies included in the review will be assessed using the tool described in Chapter 8 of the Cochrane Handbook for Systematic Reviews of Interventions [28]. An assessment of the risk of bias (high, low or unclear risk of bias) on each domain (random sequence generation, allocation concealment, blinding of participants and personnel, blinding of outcome assessment, incomplete outcome data, selective outcome reporting and other bias) will be assigned to each of the included studies and will be reported and utilised in sensitivity analysis.

### Data synthesis

#### Criteria for study data to be meta-analysed

The meta-analysis will only include those studies that report a relevant outcome measure (clinical behaviour of a health worker or patient health outcome) that can be converted into a standardised mean difference.

#### Planned approach for meta-analysis

To address the first research question (What is the effect of social norms interventions on the clinical behaviour of health workers, and resulting patient outcomes?) estimates from the individual studies will be combined using a fixed effects meta-analysis on the standardised mean difference, stratified by the 5 social norms BCTs. The research team prefers a fixed effects approach to a random effects approach in this case, since a key assumption of the latter, exchangeability, is not anticipated to hold in these trials [29]. The fixed effects analysis will yield a summary of the evidence in these trials, rather than an estimate of a common underlying treatment effect, as advocated by Higgins et al. [30]. Statistical heterogeneity will be explored and reported visually by preparing forest plots and reporting *I*^2^.

To address the second research question (Which contexts, modes of delivery and behaviour change techniques are associated with the effectiveness of social norms interventions on health worker clinical behaviour change?), if there are sufficient studies with comparable outcomes, the research team will follow steps 1 to 3:

Step 1: Explore sources of variation, using forest plots and narrative description.

Step 2: Undertake an exploratory analysis, using multivariable meta-regression to investigate sources of heterogeneity and explain variation in the results. Meta-regression is an appropriate method in which appropriate weights are assigned to studies/sub-groups. Prior to undertaking this analysis, a detailed analysis plan will be written, making explicit the sources of variation that will be investigated and our hypotheses, following the recommendations of Thompson [31]. Our categorisation of the sources of variation was informed by a meta-synthesis of audit and feedback interventions [32]. Sources of variation may include the items

1. Context

- Type of health worker (such as a family doctor, nurse, secondary care doctor or allied health professional).
- Type of behaviour (such as prescribing, hand-washing or surgical technique).
- Any targeting of participants based on baseline performance of behaviour (such as below or above average).
- Concomitant behaviour change techniques delivered alongside the social norms intervention
- Choice of reference group (such as health worker, professional body, patient or other)
- Direction of change expected (increase, decrease or maintenance of behaviour)

2. Mode of delivery

- Who delivers the intervention (such as health worker, non-health worker, patient or researcher) and whether they are internal or external to the target’s organisation.
- Frequency and intensity
- Delivery method (such as email, letter, computerised or face-to-face)

3. Social norm behaviour change technique

Step 3: Explore whether social norms interventions with particular components and concomitant behaviour change techniques are more likely to be effective. For this, the research team will consider using a multi-component-based network meta-analysis [33]. This analysis will be reliant on 2 conditions: (1) being able to identify distinct components/techniques from the published literature (2) a connected network of components/techniques. The research team will only proceed with this analysis if these two conditions hold and a pre-specified analysis plan is approved by the Study Steering Committee.

#### Additional analyses

Sensitivity analyses for the primary outcome (a) include only studies with a low risk of bias (for each separate domain and all domains), (b) include only studies where the primary outcome was reported on a continuous scale, (c) using methods of Ma et al. [34] to impute missing standard deviations, and (d) include only studies where the standard error was not imputed.

#### Planned summary if meta-analysis is not possible

If meta-analysis is not possible because of inconsistency and incomplete reporting of outcome measures, an Albatross plot, as proposed by Harrison, will be produced [35]. In an albatross plot, *p* values, ordered from extreme negative trend to extreme positive trend, are plotted against study size. Effect contours will be added to show a range of effect sizes.

#### Meta-bias

The impact of reporting bias will be minimised by performing a comprehensive search for eligible studies. Publication bias in the reported studies will be investigated using a funnel plot.

#### Confidence in cumulative evidence

The strength of the body of evidence will be assessed for the primary outcome using GRADE for each of the five BCTs and for social norms overall.

## Discussion

There are many implementation research contexts in which modification of the behaviour of health workers may have a beneficial effect on patient diagnosis, care, treatment, and on the costs of healthcare. These contexts include situations where health workers are expected to follow evidence-based professional practice such as prescribing, ordering tests, choosing treatments and adhering to guidelines. A systematic review of the evidence is needed to establish whether these interventions are effective and what factors influence their effectiveness. Limitations of the review include the following: the search strategy may not pick up every healthcare profession and could miss social norms interventions that are described using bespoke terminology, the context in which clinical behaviour takes place and the factors that influence it are complex and it is unlikely the individual studies will report these fully and consistently, the review will synthesize the results of trials with different outcome measures, by restricting the studies to those written in English, we may miss important evidence, and authors will not be contacted for intervention manuals which could lead to the omission or misclassification of some BCTs. All this could have a potential impact on interpretation of the results and recommendations for future research. This is the first systematic review, to our knowledge, that will investigate the effect of social norms interventions on health worker behaviour and resulting patient outcomes.

## Additional files

Additional file 1: PRISMA-P Checklist. (DOCX 34 kb)

Additional file 2: Final search strategies. (DOCX 36 kb)

## Data Availability

Not applicable

## Abbreviations

A&F: Audit and feedback
BCT: Behaviour change technique
EPOC: Effective Practice and Organisation of Care
GRADE: Grading of Recommendations, Assessment, Development and Evaluations
ICC: Intra-class correlation coefficient
MRC: Medical Research Council
NHS: National Health Service
RCT: Randomised controlled trial
TIDieR: Template for Intervention Description and Replication
