# Supplementary material for: The impact of social norms interventions on clinical behaviour change among health workers: protocol for a systematic review and meta-analysis
Source: Syst Rev. 2019 Jul 18;8:176. doi: 10.1186/s13643-019-1077-6 (PMC6637585; doi:10.1186/s13643-019-1077-6)
Supplement: Supplementary file 1 — PRISMA-P Checklist. (DOCX 34 kb) [file 13643_2019_1077_MOESM1_ESM.docx]

Title: The impact of social norms interventions on clinical behaviour change among health workers: protocol for a systematic review and meta-analysis.

**Authors**

Sarah Cotterill^1^ [sarah.cotterill@manchester.ac.uk](mailto:sarah.cotterill@manchester.ac.uk)

Rachael Powell^2^ [rachael.powell@manchester.ac.uk](mailto:rachael.powell@manchester.ac.uk)

Sarah Rhodes^1^ [Sarah.A.Rhodes@manchester.ac.uk](mailto:Sarah.A.Rhodes@manchester.ac.uk)

Benjamin Brown^3, 4^ [benjamin.brown@manchester.ac.uk](mailto:benjamin.brown@manchester.ac.uk)

Jane Roberts^5^ [jane.roberts@pat.nhs.uk](mailto:jane.roberts@pat.nhs.uk)

Mei Yee Tang^1^ meiyee.tang@manchester.ac.uk

Jack Wilkinson^1^ jack.wilkinson@manchester.ac.uk

Corresponding author: Sarah Cotterill, Centre for Biostatistics, Division of Population Health, Health Services Research and Primary Care, School of Health Sciences, Faculty of Biology Medicine and Health, University of Manchester, Oxford Road, Manchester, M13 9PL

^1^Centre for Biostatistics, School of Health Sciences, Faculty of Biology Medicine and Health, University of Manchester

^2^Manchester Centre for Health Psychology, Division of Psychology and Mental Health, School of Health Sciences, Faculty of Biology Medicine and Health, University of Manchester

^3^ Health e-Research Centre, Farr Institute for Health Informatics Research, Faculty of Biology Medicine and Health, University of Manchester

^4^ Centre for Primary Care, School of Health Sciences, Faculty of Biology Medicine and Health, University of Manchester

^5^ Outreach and Evidence Search Service, Library & E-learning Service, The Pennine Acute Hospitals NHS Trust, Royal Oldham Hospital.

**ABSTRACT**

Background

Health workers routinely carry out clinical behaviours, such as prescribing, test-ordering or hand-washing, which impact on patient diagnoses, care, treatment and recovery. Social norms are the implicit or explicit rules that a group uses to determine values, beliefs, attitudes and behaviours. A social norms intervention seeks to change the clinical behaviour of a target health worker by exposing them to the values, beliefs, attitudes or behaviours of a reference group or person. This study aims to find out whether or not social norms interventions are effective ways of encouraging health workers to carry out desired behaviours, and to identify which types of social norms intervention, if any, are most effective.

Methods

A systematic review will be conducted. The inclusion criteria are: a population of health professionals; a social norms intervention that seeks to change a clinical behaviour; randomised controlled trials. Searches will be undertaken in MEDLINE, EMBASE, CINAHL, British Nursing Index, ISI Web of Science, PsycINFO and Cochrane trials. Titles and abstracts will be reviewed against the inclusion criteria to exclude any that are clearly ineligible. Two reviewers will independently screen all the remaining full texts to identify relevant papers. For studies which meet our inclusion criteria, two reviewers will extract data independently, code for behaviour change techniques and assess quality using the Cochrane risk of bias tool. The primary outcome measure will be compliance with desired behaviour. To assess the effect of social norms on the behaviour of health workers we will perform random effects meta-analysis and present forest plots, stratified by behaviour change technique. We will explore sources of variation using meta-regression and may use multi-component-based network meta-analysis to explore which forms of social norms are more likely to be effective, if our data meet the necessary requirements.

Discussion: The study will provide evidence regarding the effectiveness of different methods of applying social norms to change the clinical behaviour of health professionals. We will disseminate the research to academics, health workers and members of the public, and use the findings from the review to plan future research on the use of social norms with health workers.

Systematic review registration: PROSPERO CRD42016045718. Future protocol changes will be clearly stated in PROSPERO.

Keywords

Systematic review; Meta-analysis; Social norm; Social comparison; Information about others’ approval; Credible source; Social reward; Social incentive; Feedback; Behaviour change.

**BACKGROUND**

Health workers routinely carry out behaviours which impact on patient diagnoses, care, treatment and recovery. Many of these behaviours have clear guidelines for best practice. Examples include: appropriate ordering of diagnostic tests;[1, 2] appropriate prescription of antibiotics;[3, 4] regular recall of patients with long-term conditions;[5] hand-washing;[6] choice of wound dressings.[7] Health workers face many challenges in following evidence-based professional practice. There is evidence that social influences are important in clinical practice.[8, 9]

One proposed solution has been to implement behaviour change interventions based on social or peer norms. Social norms are the implicit or explicit rules that a group uses to determine values, beliefs, attitudes and behaviours. A social norms intervention seeks to change the clinical behaviour of a target health worker by exposing them to the values, beliefs, attitudes or behaviours of a reference group or person. These social norms interventions can form part of an Audit and Feedback initiative,[10-12] or may be developed as another behaviour change intervention.[13] These are interventions with reach that can be implemented routinely across multiple health workers and settings at low cost, so the absolute gain can be very large. We use the term target to refer to a health worker who is targeted by social norms interventions, with a view to changing their clinical behaviour. We use the term reference group or reference person to mean a person or group of people used as a reference category in a social norms intervention. For the purposes of our review, we anticipate that reference categories will include people with the same profession or occupation as the target, people employed by the same organisation as the target, people who deliver, administer, manage, commission or make policy on health services, or professional bodies such as royal colleges, trade unions. It is possible that some studies will use social norms approaches where the reference group is not taken from the above list (such as credible source from a celebrity or exposing the target’s behaviour to patients). We will include in the review papers with any type of reference group.

The ability of social norms to affect behaviour has been considered within several behaviour change theories and theoretical frameworks. For example, ‘subjective norm’ is a construct within the Theory of Planned Behaviour,[14] which describes an individual’s perceptions of whether valued others think one should perform a behaviour, combined with one’s motivation to comply with others’ beliefs. The Theory of Normative Social Behaviour [15] proposes that behaviour can be changed through normative mechanisms, and has made distinctions between descriptive norms (beliefs concerning the prevalence of a behaviour) and injunctive norms (beliefs concerning what one feels they ought to do based on others’ expectations – social approval). Further, the ‘social influences’ domain of the Theoretical Domains Framework [16] also includes several normative constructs: social norms, social comparisons, and group norms. We will include studies based on either descriptive norms or injunctive norms messages. A descriptive norms message provides the target with information about the behaviour of others in the reference group. Examples of descriptive norms interventions include: giving the target information about the behaviour of a reference person or group; or comparison of the target’s behaviour with the behaviours of a reference person or group. An injunctive norms message provides the target with information about the values, beliefs or attitudes of the reference group, conveying social approval or disapproval. Examples of injunctive norms interventions include: providing the target with information about whether the behaviour has the approval/disapproval of the reference group or person; exposure (actual or promised) of the target’s behaviour to a reference group; praise, commendation, applause or thanks (actual or promised) from a reference group or person.

The Behaviour Change Technique Taxonomy v1 is a list of 93 distinct behaviour change techniques (BCTs) which are used in behaviour change interventions.[17] The BCT Taxonomy includes five BCTs which we believe involve social norms: social comparison, information about others’ approval, credible source, social reward and social incentive.[17] We have chosen to define social norms in terms of the BCT taxonomy v1 because, based on international consensus, it aims to define and label all active ingredients of interventions, including social norms. It incorporates previous behaviour change taxonomies and has involved significant effort from leaders in the field and considerable investment from the MRC and NIHR in developing the taxonomy. We believe this to be the most reliable tool currently available that can define BCTs. We have selected the five BCTs that we consider have a social norms element to them, and we have discussed this selection carefully, both within the research team and with our steering group of international experts. We are open to the possibility that studies may be eligible for the review that test social norms interventions but do not incorporate one of these five identified BCTs.

Health workers frequently receive Audit and Feedback (A&F), which involves ‘providing a recipient with a summary of their performance over a specified period of time’.[10 p 1] Social norms interventions are sometimes included as one component of A&F, such as when the health worker is shown information about their own performance and also a comparison with their peers.[11, 12] A&F has already been shown to be effective in changing health worker behaviour, but with large variation in outcomes depending on the context and the intervention design.[18] There is a need to understand the ingredients for successful A&F,[10, 19] and the effects or mechanisms of the social norms constituents of A&F have been identified in a recent systematic review as topics for further research.[10] Our review will contribute to this important research agenda by systematically examining the evidence for using social norms BCTs with health workers.

***Aims***

The overall aim is to conduct a systematic review to assess, among health workers, the impact of social norms BCTs, compared to alternative interventions, no intervention or comparison of one or more social norms BCTs on compliance with evidence-based professional practice. The review will address two research questions:

1. What is the effect of social norms interventions on the clinical behaviour of health workers, and resulting patient outcomes?
2. Which contexts, modes of delivery and behaviour change techniques are associated with the effectiveness of social norms interventions on health worker clinical behaviour change?

**METHODS**

This protocol follows the PRISMA-P reporting guidelines for systematic reviews [20] (PRISMA-P checklist included as additional file)

**Eligibility criteria**

The inclusion criteria for the review are: a population of health professionals; a social norms intervention that seeks to change a clinical behaviour; and the study type is a randomised controlled trial.

***Population***

The population of interest is health workers and managers. Student health workers will be included, but only if the study is in a healthcare setting. Any healthcare setting will be eligible, including care homes, nursing homes and patients’ own homes. Interventions in educational establishments or simulated environments will not be eligible.

***Interventions***

The systematic review will focus on social norms interventions, defined as interventions seeking to change the clinical behaviour of a target health worker by exposing them to the values, beliefs, attitudes or behaviours of a reference group or person. We have selected five BCTs (from the BCT Taxonomy v1) that we consider have a social norms element to them (6.2. Social comparison; 6.3. Information about others’ approval; 9.1 Credible source; 10.4 Social reward; 10.5 Social incentive), but we are open to the possibility that studies may be eligible for the review that test social norms interventions without using one of the five BCTs. Three BCTS are used unchanged in this review (Social comparison, information about others’ approval and credible source). Two BCTs have been adapted slightly for clarity: the definitions of social reward as ‘verbal or non-verbal reward’ and social incentive as ‘verbal or non-verbal incentive’ are insufficient to distinguish a ‘social’ reward incentive from other types of reward or incentive. Further, in the present study, we are interested in only those social rewards or incentives that rely on social norms. We define social reward and incentive as involving praise, commendation, applause or thanks, all of which are injunctive norms messages, providing the target with information about the values, beliefs or attitudes of the reference group, conveying social approval or disapproval (table 1).

(insert table 1 about here)

Included studies must state a behaviour that is being targeted for change. By definition, BCTs relate to behaviour(s): ‘a single action or sequence of actions’. Either the ‘performance of wanted behaviour(s) and/or inhibition (non-performance) of unwanted behaviour(s)’ might be addressed by a BCT.[17] [Detail/quotes are from Electronic Supplementary Materials, p1.] We will report the number of studies which would otherwise meet our inclusion criteria, but do not mention a target behaviour.

The format of the behaviour change intervention may be letter, electronic or verbal. It may be delivered once only, repeated over time, or delivered in a timely fashion on occasions when the behaviour is expected to be performed. For example, an intervention to reduce prescribing of antibiotics by family doctors might be delivered once only, by regular weekly email, or by a computerised reminder when a relevant disease code is entered into the practice computer system.

***Comparators***

We anticipate finding a range of comparators, including alternative intervention, no intervention or comparison of one or more social norms BCTs (table 2).

(insert table 2 about here)

***Study designs***

The systematic review will only include randomised controlled trials (RCTs), of any design (cluster, factorial, parallel, cross-over and stepped wedge). The justification for restricting the review to RCTs is that the review is concerned with the effectiveness of social norms, and randomised controlled trials are the best method for assessing the effectiveness of an intervention. We will include both published and unpublished research. Studies must be reported in English because the research team has no resource for translation from other languages.

**Information Sources and search strategy**

The search strategy was developed collaboratively between the researchers and the information specialist in our review team. The search targeted databases relevant to health, social and behavioural science, without restriction on dates: MEDLINE, Ovid; EMBASE, Ovid; CINAHL, Ebsco; British Nursing Index; ISI Web of Science; PsycINFO and Cochrane trials. The search was structured to find the populations (health workers), interventions (social norms) and study types (RCTs) of interest. The population search terms were based on health worker search terms from previous reviews, supplemented by a list of health worker roles from a local NHS trust and review by the study team. Intervention search terms were based on the descriptions of social norms BCTs in the BCT taxonomy v1, audit and feedback search terms, and theories relevant to social norms. RCT search filters are those described in Chapter 6.4,11 of the Cochrane Handbook for Systematic Reviews of Interventions.[21] The search terms were developed by the study team and used to develop searches in MEDLINE. They were then reviewed by the project team and translated into the other databases using the appropriate controlled vocabulary as applicable. The final search strategy was reviewed by the Management Group and Steering Group. Searches were completed between 4^th^ and 19^th^ July 2018. The MEDLINE search is available within our Prospero registration[22] and as Additional file 1.

**Data collection**

***Data management***

Covidence will be utilised as a management tool for the review. <https://www.covidence.org/>

***Study selection process***

One reviewer will independently screen the titles and abstracts and exclude studies which obviously do not meet inclusion criteria. A second reviewer will independently screen a sample of 20% of records. If there is any difference of opinion at the title/abstract stage, the reviewers will err on the side of inclusion. The number of cases of disagreement will be reported. If the level of disagreement is over 10%, all the records will be double screened. Two reviewers will independently screen the full texts and apply the eligibility criteria. If there is any difference of opinion, the two screeners will discuss, and if they cannot agree, the text will be reviewed by a third member of the research team, or resolved at a project team meeting if required.

***Data collection process***

Data from included studies will be extracted independently by two researchers. A data collection template, based on the Cochrane EPOC data collection form[23] has been developed,[22] which will ensure that (where available) information on population and setting, methods, participants, interventions, controls, outcomes, results and applicability is collected. Discrepancies between reviewers will be resolved through discussion between the two researchers, by involving a third member of the research team, or discussion within the full project team meeting where necessary. Interventions will be described using relevant items from the TIDieR checklist [24] and specific behaviour change techniques will be classified using the behaviour change technique taxonomy, version 1.[17]

***Unit of analysis issues***

If any of the studies in the review are cluster randomised trials, summary measures (e.g. means, odds ratios) and adjusted standard errors will be extracted from appropriately analysed trials. Where necessary adjustments for clustering will be made, using the ICC (intra-class correlation coefficient). If more than one comparison from a study with more than two arms is eligible for the same comparison, the number of health workers in the shared arm will be adjusted to avoid double counting. The adjustment will be done by dividing the number of health workers in the shared arm approximately evenly among the comparisons.

***Missing data***

The research team will search for companion papers (by author searching and citation searching) and/or contact trial authors once to obtain missing information under the following circumstances:

1. Where there is insufficient information in the full trial report to establish whether or not the trial meets our inclusion criteria
2. Where it is clear that our primary outcome measure was measured but insufficient information was reported to establish the number of participants and/or summary measures.
3. Where the intervention descriptions are insufficiently clear to determine whether or not the trial meets our inclusion criteria.

The research team will impute estimates of standard deviations where necessary (after contacting authors) using standard deviations from other similar studies (same target behaviour, same setting) that use the same type of outcome. Where necessary, for cluster randomised trials, a value of the ICC from similar studies (same target behaviour, same outcome measure, same setting) will be imputed.

***Piloting***

All processes for screening, extraction, BCT coding and assessment of bias will be piloted within the team prior to implementation

***Coding of Behaviour Change Techniques***

Specific behaviour change techniques will be coded independently by two researchers, using the behaviour change technique taxonomy v1.[17] The reliability of identifying and coding behaviour change techniques from intervention descriptions has been assessed.[25] This research assessed the reliability of judgements between different coders and across time using the prevalence and bias-adjusted kappa (PABAK) statistic. Overall, there were high rates of reliability between coders. Of the five BCTs relevant to this review, three of the techniques (social comparison, information about others’ approval, social incentive) were assessed in a high number of studies (6 to 20 studies) and were found to have high inter-rater reliability (over 0.7). Social reward was assessed in only one study but the reliability was high (1.0). Credible source was found in a high number of studies, but found to have lower reliability (0.4) (table 3).

(insert table 3 about here)

All coders will be trained in coding of behaviour change techniques, using the on-line training provided by the authors of the BCT taxonomy (<http://www.bct-taxonomy.com/> ), which is required to meet acceptable standards of competence. The online training has been shown to improve agreement with expert consensus, confidence for BCTs assessed and coding competence [26]. Additionally they have attended a workshop facilitated by co-applicant RP and steering group member MJ. The level of agreement on the coding of BCTs between two coders will be reported, using a PABAK statistic.

***Outcomes and prioritisation***

The primary outcome for this review is compliance of the health worker with the desired clinical behaviour (e.g. rate of antibiotic use) at 6 months post randomisation. Six months post randomisation was chosen to identify a common time point from randomisation and there is the potential for interventions to run over several months. Researchers will extract details of the outcome closest to 6 months post randomisation (e.g. mean and standard deviation or proportion). Other time points will be noted for inclusion in the description of studies and the team may consider conducting analysis using earlier/later time points, where reported.

It is likely that different studies will use different outcome measures as they will be measuring different behaviours and using different methods to assess those behaviours. The research team will convert any observed measure of health worker behaviour into a standardised mean difference between groups in terms of compliance with the desired behaviour. Examples include the mean number of times the behaviour was performed per worker or the mean rate of behaviour (e.g. rate of antibiotic items dispensed per 1000 population). It is possible that compliance will be reported as a binary outcome, for example compliance v non-compliance on a single occasion, e.g. attendance a training session. The methods of Chin 2000 will be adopted to convert binary outcomes to standardised mean differences with associated standard errors using the formula:[27]

                               
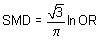


If several measures of compliance are reported in a trial the following criteria will be used to select the outcome for the primary analysis, in decreasing order of importance: (a) observed measure rather than self-report; (b) continuous measure (c) final score rather than change from baseline or percentage change (d) described as the primary outcome (e) used to calculate the sample size (f) reported first.

A secondary outcome for the review is any patient outcomes which are likely to result from targeting the health worker behaviour.

***Risk of bias in individual studies***

Two reviewers will independently assess the risk of bias of each study; discrepancies will be resolved by discussion between the two reviewers, by involving a third reviewer, or by discussion within the project team, if needed. The risk of bias for each main outcome in all studies included in the review will be assessed using the tool described in Chapter 8 of the Cochrane Handbook for Systematic Reviews of Interventions.[28] An assessment of the risk of bias (high, low or unclear risk of bias) on each domain (random sequence generation, allocation concealment, blinding of participants and personnel, blinding of outcome assessment, incomplete outcome data, selective outcome reporting, and other bias) will be assigned to each of the included studies, and will be reported and utilised in sensitivity analysis.

**Data synthesis**

***Criteria for study data to be meta-analysed***

The meta-analysis will only include those studies that report a relevant outcome measure (clinical behaviour of a health worker or patient health outcome) that can be converted into a standardised mean difference.

***Planned approach for meta-analysis***

To address the first research question (*What is the effect of social norms interventions on the clinical behaviour of health workers, and resulting patient outcomes?)* estimates from the individual studies will be combined using a fixed effects meta-analysis on the standardised mean difference, stratified by the 5 social norms BCTs. The research team prefer a fixed effects approach to a random effects approach in this case, since a key assumption of the latter, exchangeability, is not anticipated to hold in these trials (Poole and Greenland, 1999). The fixed effects analysis will yield a summary of the evidence in these trials, rather than an estimate of a common underlying treatment effect, as advocated by Higgins et al. [29] Statistical heterogeneity will be explored and reported visually by preparing forest plots and reporting I^2^.

To address the second research question (*Which contexts, modes of delivery and behaviour change techniques are associated with the effectiveness of social norms interventions on health worker clinical behaviour change)*, if there are sufficient studies with comparable outcomes, the research team will follow steps 1 to 3:

Step 1: Explore sources of variation, using forest plots and narrative description.

Step 2: Undertake an exploratory analysis, using multivariable meta-regression to investigate sources of heterogeneity and explain variation in the results. Meta-regression is an appropriate method in which appropriate weights are assigned to studies/sub-groups. Prior to undertaking this analysis a detailed analysis plan will be written, making explicit the sources of variation that will be investigated, and our hypotheses, following the recommendations of Thompson 2002.[30] Our categorisation of the sources of variation will be informed by an on-going meta-synthesis of qualitative studies of audit and feedback interventions, currently being completed by one of our co-applicants (BB). Sources of variation may include:

i) Context

- Type of health worker (such as family doctor, nurse, secondary care doctor or allied health professional).
- Type of behaviour (such as prescribing, hand-washing or surgical technique).
- Any targeting of participants based on baseline performance of behaviour (such as below or above average).
- Concomitant behaviour change techniques delivered alongside the social norms intervention
- Choice of reference group (such as health worker, professional body, patient or other)
- Direction of change expected (increase or decrease or maintenance of behaviour)

ii) Mode of delivery

- Who delivers the intervention (such as health worker, non-health worker, patient or researcher) and whether they are internal or external to the target’s organisation.
- Frequency and intensity
- Delivery method (such as email, letter, computerised or face-to-face)

iii) Social norm behaviour change technique

Step 3: Explore whether social norms interventions with particular components and concomitant behaviour change techniques are more likely to be effective. For this the research team will consider using a multi-component-based network meta-analysis.[31] This analysis will be reliant on 2 conditions: (i) being able to identify distinct components/techniques from the published literature (ii) a connected network of components/techniques. The research team will only proceed with this analysis if these two conditions hold and a pre-specified analysis plan is approved by the review Steering Committee.

***Additional analyses***

Sensitivity analyses for the primary outcome: a) Include only studies with a low risk of bias (for each separate domain and all domains); b) Include only studies where the primary outcome was reported on a continuous scale; c) Using methods of Ma at al. [32] to impute missing standard deviations d) Include only studies where the standard error was not imputed.

***Planned summary if meta-analysis is not possible***

If meta-analysis is not possible because of inconsistency and incomplete reporting of outcome measures, an Albatross plot, as proposed by Harrison, will be produced.[33] In an albatross plot p-values, ordered from extreme negative trend to extreme positive trend are plotted against study size. Effect contours will be added to show a range of effect sizes.

***Meta-bias***

The impact of reporting bias will be minimised by performing a comprehensive search for eligible studies. Publication bias in the reported studies will be investigated using a funnel plot.

***Confidence in cumulative evidence***

The strength of the body of evidence will be assessed for the primary outcome using GRADE for each of the five BCTs and for social norms overall.

**DISCUSSION**

There are many implementation research contexts in which modification of the behaviour of health workers may have a beneficial effect on patient diagnosis, care, treatment, and on the costs of healthcare. These contexts include situations where health workers are expected to follow evidence-based professional practice such as prescribing, ordering tests, choosing treatments and adhering to guidelines. A systematic review of the evidence is needed to establish whether these interventions are effective, and what factors influence their effectiveness. Limitations of the review include: the search strategy may not pick up every healthcare profession and could miss social norms interventions that are described using bespoke terminology; the context in which clinical behaviour takes place and the factors that influence it are complex and it is unlikely the individual studies will report these fully and consistently; the review will synthesize the results of trials with different outcome measures; by restricting the studies to those written in English we may miss important evidence; authors will not be contacted for intervention manuals which could lead to the omission or misclassification of some BCTs. All this could have a potential impact on interpretation of the results and recommendations for future research. This is the first systematic review, to our knowledge, that will investigate the effect of social norms interventions on health worker behaviour and resulting patient outcomes.

**List of abbreviations**

A&F Audit and Feedback

BCT Behaviour Change Technique

EPOC Effective Practice and Organisation of Care

GRADE Grading of Recommendations, Assessment, Development and Evaluations

ICC Intra-class correlation coefficient

MRC Medical Research Council

NHS National Health Service

RCT Randomised Controlled Trial

TIDieR Template for Intervention Description and Replication

**Declarations**

***Ethics approval and consent to participate***

Not applicable

***Consent for publication***

Not applicable

***Availability of data and material***

Not applicable

***Competing interests***

The authors declare that they have no competing interests.

***Funding***

This report is independent research funded by the National Institute for Health Research (Health Services and Delivery Research, 17/06/06 - The impact of social norms interventions on clinical behaviour change among health workers: a systematic review). The views expressed in this publication are those of the author(s) and not necessarily those of the NHS, the National Institute for Health Research, the Department of Health or the sponsor (The University of Manchester).

***Authors' contributions***

SC is the guarantor. SC drafted the protocol. SC, RP, SR and BB wrote the funding bid on which this protocol is based. SC, RP, SR, BB, MYT and JW contributed to the development of the selection criteria, the risk of bias assessment strategy and data extraction criteria. SC, RP, MYT, JR developed the search strategy. SR and JW provided statistical expertise. All authors read, provided feedback and approved the final manuscript.

***Acknowledgements***

The authors thank the experts on our study steering group who have provided valuable advice and guidance: Sofia Dias, Robbie Foy, Marie Johnston and Manoj Mistry.

References

1. Hamilton W, Watson J, Round A: **Investigating fatigue in primary care.** *British Medical Journal* 2010, **341**.

2. NICE: **Cardiovascular disease: risk assessment and reduction, including lipid modification Clinical guideline [CG181] Published July 2014, updated September 2016 nice.org.uk/guidance/cg181.** 2014.

3. Davies SC, Fowler T, Watson J, Livermore DM, Walker D: **Annual Report of the Chief Medical Officer: infection and the rise of antimicrobial resistance.** *Lancet* 2013, **381:**1606-1609.

4. Hawker JI, Smith S, Smith GE, Morbey R, Johnson AP, Fleming DM, Shallcross L, Hayward AC: **Trends in antibiotic prescribing in primary care for clinical syndromes subject to national recommendations to reduce antibiotic resistance, UK 1995-2011: analysis of a large database of primary care consultations.** *J Antimicrob Chemother* 2014, **69:**3423-3430.

5. NICE: **Guidance. Type 2 diabetes in adults: management. Published: 2 December 2015. nice.org.uk/guidance/ng28.**

6. Loveday HP, Wilson JA, Pratt RJ, Golsorkhi M, Tingle A, Bak A, Browne J, Prieto J, Wilcox M: **epic3: National Evidence-Based Guidelines for Preventing Healthcare-Associated Infections in NHS Hospitals in England.** *Journal of Hospital Infection*, **86:**S1-S70.

7. NICE advice: **Chronic wounds: advanced wound dressings and antimicrobial dressings. Evidence summary, Published: 30 March 2016 nice.org.uk/guidance/esmpb2.**

8. Whiting P, Toerien M, de Salis I, Sterne JAC, Dieppe P, Egger M, Fahey T: **A review identifies and classifies reasons for ordering diagnostic tests.** *Journal of Clinical Epidemiology* 2007, **60:**981-989.

9. van der Weijden T, van Bokhoven MA, Dinant G-J, van Hasselt CM, Grol RPTM: **Understanding laboratory testing in diagnostic uncertainty: a qualitative study in general practice.** *The British Journal of General Practice* 2002, **52:**974-980.

10. Ivers NM, Sales A, Colquhoun H, Michie S, Foy R, Francis JJ, Grimshaw JM: **No more 'business as usual' with audit and feedback interventions: towards an agenda for a reinvigorated intervention.** *Implement Sci* 2014, **9:**14.

11. Ivers NM, Tu K, Young J, Francis JJ, Barnsley J, Shah BR, Upshur RE, Moineddin R, Grimshaw JM, Zwarenstein M: **Feedback GAP: pragmatic, cluster-randomized trial of goal setting and action plans to increase the effectiveness of audit and feedback interventions in primary care.** *Implementation Science* 2013, **8:**142.

12. Carney PA, Abraham L, Cook A, Feig SA, Sickles EA, Miglioretti DL, Geller BM, Yankaskas BC, Elmore JG: **Impact of an educational intervention designed to reduce unnecessary recall during screening mammography.** *Academic Radiology* 2012, **19:**1114-1120.

13. Huis A, Schoonhoven L, Grol R, Donders R, Hulscher M, van Achterberg T: **Impact of a team and leaders-directed strategy to improve nurses' adherence to hand hygiene guidelines: a cluster randomised trial.** *Int J Nurs Stud* 2013, **50:**464-474.

14. Ajzen I: **The theory of planned behavior.** *Organizational Behavior and Human Decision Processes* 1991, **50:**179-211.

15. Rimal RN, Real K: **How Behaviors are Influenced by Perceived Norms:A Test of the Theory of Normative Social Behavior.** *Communication Research* 2005, **32:**389-414.

16. Cane J, O’Connor D, Michie S: **Validation of the theoretical domains framework for use in behaviour change and implementation research.** *Implementation Science* 2012, **7:**37.

17. Michie S, Richardson M, Johnston M, Abraham C, Francis J, Hardeman W, Eccles MP, Cane J, Wood CE: **The behavior change technique taxonomy (v1) of 93 hierarchically clustered techniques: building an international consensus for the reporting of behavior change interventions.** *Ann Behav Med* 2013, **46:**81-95.

18. Ivers N, Jamtvedt G, Flottorp S, Young JM, Odgaard-Jensen J, French SD, O'Brien MA, Johansen M, Grimshaw J, Oxman AD: **Audit and feedback: effects on professional practice and healthcare outcomes.** *Cochrane Database Syst Rev* 2012, **6:**CD000259.

19. Gardner B, Whittington C, McAteer J, Eccles MP, Michie S: **Using theory to synthesise evidence from behaviour change interventions: the example of audit and feedback.** *Soc Sci Med* 2010, **70:**1618-1625.

20. Moher D, Shamseer L, Clarke M, Ghersi D, Liberati A, Petticrew M, Shekelle P, Stewart LA: **Preferred reporting items for systematic review and meta-analysis protocols (PRISMA-P) 2015 statement.** *Syst Rev* 2015, **4:**1.

21. Higgins JPT GSe: *Cochrane Handbook for Systematic Reviews of Interventions Version 5.1.0 [updated March 2011]. .* The Cochrane Collaboration; 2011.

22. Cotterill S, Powell R, Rhodes S, Roberts J, Tang MY, Wilkinson J: **The impact of social norms interventions on clinical behaviour change among health workers: protocol for a systematic review. PROSPERO 2016 CRD42016042718 Available from: http://www.crd.york.ac.uk/PROSPERO/display_record.php?ID=CRD42016042718.**

23. Effective Practice and Organisation of Care (EPOC): **Data collection form: EPOC resources for review authors.** Oslo: Norweigian Knowledge Centre for the Health Services; 2013.

24. Hoffmann TC, Glasziou PP, Boutron I, Milne R, Perera R, Moher D, Altman DG, Barbour V, Macdonald H, Johnston M, et al: **Better reporting of interventions: template for intervention description and replication (TIDieR) checklist and guide.** *BMJ* 2014, **348:**g1687.

25. Abraham C, Wood CE, Johnston M, Francis J, Hardeman W, Richardson M, Michie S: **Reliability of Identification of Behavior Change Techniques in Intervention Descriptions.** *Ann Behav Med* 2015, **49:**885-900.

26. Wood CE, Richardson M, Johnston M, Abraham C, Francis J, Hardeman W, Michie S: **Applying the behaviour change technique (BCT) taxonomy v1: a study of coder training.** *Translational Behavioral Medicine* 2015, **5:**134-148.

27. Chinn S: **A simple method for converting an odds ratio to effect size for use in meta-analysis.** *Statistics in Medicine* 2000, **19:**3127-3131.

28. Higgins JPT AD, Sterne JAC (editors). **Chapter 8: Assessing risk of bias in included studies.** In *Cochrane Handbook for Systematic Reviews of Interventions Version 510 (updated March 2011).* Edited by Higgins JPT GS: The Cochrane Collaboration; 2011

29. Higgins JPT, López-López JA, Becker BJ, Davies SR, Dawson S, Grimshaw JM, McGuinness LA, Moore THM, Rehfuess EA, Thomas J, Caldwell DM: **Synthesising quantitative evidence in systematic reviews of complex health interventions.** *BMJ Global Health* 2019, **4:**e000858.

30. Thompson SG, Higgins JP: **How should meta-regression analyses be undertaken and interpreted?** *Stat Med* 2002, **21:**1559-1573.

31. Caldwell DM, Welton NJ: **Approaches for synthesising complex mental health interventions in meta-analysis.** *Evidence Based Mental Health* 2016.

32. Ma J, Liu W, Hunter A, Zhang W: **Performing meta-analysis with incomplete statistical information in clinical trials.** *BMC Medical Research Methodology* 2008, **8:**56.

33. Harrison S: **Albatross plots, a novel method of displaying data for otherwise un-combinable studies.** In *Young Statisticians Meeting*. Cardiff University, UK; 2015.
